# Nexus of Emotional Intelligence and Learning Outcomes: A Cross-Country Study of China and Pakistan Higher Educational Institutes

**DOI:** 10.3390/ijerph192316215

**Published:** 2022-12-04

**Authors:** Zahid Shafait, Jiayu Huang

**Affiliations:** 1 College of Teacher Education, Zhejiang Normal University, Jinhua 321004, China; 2 National Governance Research Institute, School of Marxism, Zhejiang Normal University, Jinhua 321004, China

**Keywords:** China, Pakistan, emotional intelligence, learning outcomes, self-directed learning, knowledge management processes, creative performance

## Abstract

The purpose of this empirical study is to investigate the effects of emotional intelligence (EI) on learning outcomes (e.g., social, cognitive, and self-growth outcomes) and satisfaction with the university experience of academics and administrative staff at Chinese and Pakistani research universities. This study also investigates the mediation of self-directed learning (personal autonomy, personal responsibility, and personal growth) and knowledge management processes (KMPs) concerning the relationship between EI and learning outcomes. Moreover, this study explores the relationship between learning outcomes and creative performance (creative self-efficacy and leadership/supervisor support). The survey method was considered appropriate for the data collection and was completed simultaneously through paper and electronic mediums. The partial least squares structural equation modeling (PLS-SEM) method with a measurement assessment, structural assessment, mediation, and multi-group analysis was applied to a sample of 729 academics and administrative staff from Chinese and Pakistani research universities. A few dissimilarities surfaced with regard to EI and learning outcomes while evaluating the higher education institutions (HEIs) from both countries. Moreover, an indirect relation between EI and learning outcomes was established via self-directed learning and KMPs. Lastly, the intended direct statistical association between learning outcomes and creative performance was also documented. This study may serve as an initiative to equate and differentiate EI in relation to learning outcomes and creative performance among higher education professionals in China and Pakistan. The considered framework is novel and supports both EI and learning outcomes while adhering to the perceived value of the two adjacent regions.

## 1. Introduction

Over the past decades, Western HEIs have had a dominant influence worldwide, and Asian countries have benefited substantially from the established standards and protocols of Western pedagogic patterns. Nevertheless, Western/international supremacy in terms of education control and provision has consistently been challenged by the Asian region. Hence, many Asian countries are eager to become the “Asian tiger” in the field of education [1]. Therefore, the educational sector of Asia, especially that of China and Pakistan, has progressed significantly to develop advanced skills and knowledge to become strategically competitive, promptly banish pedagogic inconsistencies, and exploit learning opportunities as they materialize [2]. Moreover, continued survival and growth are generally achieved and maintained through the strategic utilization of scarce, intangible, and firm-specific resources, which include emotional intelligence (EI) training and development within the immediate workforce [3]. The ability to ascertain, express (personal emotions), comprehend, and manage others’ emotions is known as EI [4]. Similarly, HEIs’ personnel with a higher level of EI exhibit superior psychological well-being and comprehend and practice enhanced public and interpersonal relationships, which are vital to their learning outcomes [5].

Therefore, higher education institutions (HEIs) should expand their competitive edge to increase their globalization status [4]. For example, China had developed exceptional growth in higher education throughout the five years prior to 2018. According to the Ministry of Education of China [6], the total number of higher education institutions in China reached 8284. The gross enrollment rate of higher education reached 45.7%, increasing 3% from the previous year, reaching 27.6 million. Likewise, in 2016, China invited approximately 442,773 (an increase of 11.35% as compare to 2015) global scholars to its HEIs from 205 countries. Furthermore, 15,000 foreigners joined Chinese HEIs as academics or administrative personnel in 2013 [7]. Likewise, according to the Economic Survey of Pakistan [8] in 2018, the country witnessed an upward growth, though minor, in higher education over the prior five-year period. According to the survey, in 2018, the number of higher education schools in Pakistan was 194; teachers hired/enrollment were 56,900. In 2018, student enrollment for higher education in Pakistan reached 1.576 million as compared to 2017, when HEIs attracted 1.463 million students.

Additionally, HEIs’ activities in China and Pakistan are currently in their infancy phase in relation to research as explained by Latif [2] and Turner and Acker [9]. Possible explanations for this outcome may be attributed to the following: lack of students’ inclination and motivation for research and developmental activities; lack of teachers’ reformation in terms of training and development; educational inequality in terms of gender and regions; lack of proper policy formulations and implementations in HEIs for research and learning; outdated curriculums; corruption; poor management and supervision that hinder the expected outcomes; lack of research interests individually and institutionally; and poor infrastructure and facilities for research and developmental activities [10,11]. Even so, compared to 65.5% in 1982, China has made substantial progress, securing a 95.1% literacy rate in 2018, while Pakistan continued to lag well behind with a literacy rate of 62.3% in 2018/19 [8]. Moreover, the growing attraction of HEIs in China and Pakistan has transformed them from elite to mass institutions, which has fostered numerous challenges for HEIs [12,13]. HEIs’ personnel are now confronted with increased workloads and, thus, may experience significant pressure from teaching, conducting research, publishing academic papers, and professional status evaluations. Therefore, by introducing EI and constant encouragement (e.g., training and development), institutions have helped ease some of these situations [14].

Contemporary thoughts on EI and its respective effect on several phases of the professional sphere have encouraged researchers to evaluate and investigate EI across organizations and countries, including China and Pakistan, with a focus on its importance in the pedagogic sector [3,4]. Moreover, HEIs’ professionals require skills that are grounded in EI models. These include, but are not limited to the following: cognitive abilities or personality traits; awareness of personal emotions; decisiveness; compassion; problem-solving ability; self-control; openness; societal obligation; elasticity; inspiration; communication; collaboration; and motivation [5,15]. However, very few studies acknowledge the links between personnel in HEIs and EI, even though it is an important factor [3,16]. Nevertheless, when HEIs offer a positive climate that facilitates the mastery of emotional management, it strengthens the personnel’s experience to cope with high-pressure situations while facilitating knowledge acquisition. Knowledge acquisition is also further facilitated based on emotional responses [4]. Furthermore, numerous studies in the education sector have discovered a positive relationship between the following: EI and psychological well-being; social functioning and the quality of interpersonal relationships; gracing unseen occasions; health indicators; job performance; and learning outcomes [4,5,17,18,19,20].

HEIs’ personnel and EI have a distinctive symbiotic relationship; however, emotions have been relatively neglected in educational research, which is alarming, as emotions are at the core of learning [3]. Emotions stimulate learners’ attention, which extends the learning process and outcomes while affecting what is learned and, more importantly, retained [21]. Studies across the disciplines, including HEIs, have revealed that emotions play an important role in learning outcomes [5,22,23]. Furthermore, HEIs’ personnel, in conjunction with EI, stimulate colleagues’ abilities to enhance learning outcomes by utilizing effective emotional management protocols [24]. Additionally, HEIs from China and Pakistan have showcased their contribution to EI through enhanced learning outcomes [25,26]. Likewise, in a recent study by Shafait [22] emphasized that future researchers should conduct cross-cultural studies of Chinese and Pakistani HEIs’ professionals in relation to their EI and learning outcomes.

In the context of Chinese and Pakistani HEIs, self-directed learning is considered a vital force for professional development [3,5]. Therefore, self-directed learning is especially appreciated in HEIs, because it supports personal autonomy, personal responsibility, and personal growth, exemplifying the fundamental standards of higher education [27]. Self-directed learning is a combination of standards and initiatives intended for learning, such as establishing the precise specification of learning prerequisites; formulating learning objectives; identifying social and quantifiable resources for anticipated learning; selecting applicable learning approaches and their implementation; and evaluating learning outcomes with or without assistance from others [28]. Even though self-directed learning in HEIs has been extensively investigated, the research regarding facilitative factors of self-directed learning in HEIs is rather sporadic [5]. However, this study intends to fill this void by investigating and analyzing EI in HEIs. Likewise, being a self-directed learner could prove challenging, as personnel in HEIs experience and manage diverse emotions such as pessimism, misperception, hindrance, and disappointment, thus paving the way toward developing a novel learning experience [29]. Numerous researches have similarly established positive associations among self-directed learning and multiple learning outcomes [3,5]. For example, Lounsbury, Levy [30] posited that self-directed learning remained expressly interconnected with multiple cognitive ability processes. These include abilities that are correlated to oral proficiency, mathematical proficiency, theoretical skills, and pragmatic and rational thinking. Similarly, self-directed learning experience improves the confidence of personnel in HEIs, motivating them to learn and hence improving their critical thinking skills and the quality of understanding various assigned tasks [3].

It seems as if there is a general consensus between the resource- and knowledge-based perspectives that organizations share in attempting to sustain a competitive lead, which may also result in manipulating available knowledge and skills for value conception [31]. However, irrespective of the enhanced importance of the knowledge management processes (KMPs) in HEIs, the implemented knowledge management policies remain insufficient, especially in developing countries such as China and Pakistan [3,32]. By employing the Goleman [33] model of EI, Karkoulian [34] found a positive linkage between EI and KMPs. Goh and Lim [35] later endorsed the Karkoulian [34] study. These previous studies by Karkoulian [34] and Goh and Lim [35] were conducted in commercial sectors; however, this study investigates the various phenomena in HEIs. Knowledge management and knowledge workers are both dependent upon each other [31]. Given the knowledge-based settings, a knowledge worker seeks emotionally intelligent peers to develop mechanisms for knowledge management. Moreover, these individuals can be assessed according to the best knowledge workers an entity may have available who also possess an adequate level of EI [3]. These authors have also recently examined EI and KMPs directly. Furthermore, Bouncken and Kraus [36] proffered that knowledge is an important factor that contributes to technology, innovation, design, and market success in businesses. This could be acknowledged as similar advantages that can be retained by HEIs. Furthermore, certain studies have specifically examined EI through knowledge sharing and knowledge transfer; however, these studies suggested evaluating KMPs in knowledge-based organizations [35,37].

Furthermore, knowledge and learning, whichever method is adopted, share a strong interdependency for growth. Ramachandran [38] investigated HEIs and established a link between knowledge management and learning outcomes. Moreover, Leal-Rodríguez [39] investigated the relationship between KMP and learning outcomes within the commercial sector, which differs from this study that investigated HEIs with the stated variables.

Currently, HEIs are channeling a creative performance myth among their immediate beneficiaries. Hence, creative performance confines HEIs to acquire and maintain sustainable performance in order to maintain a competitive advantage [3]. Additionally, Shalley [40] argued that dynamic work assignments necessitate increased creative problem solving; therefore, personnel are expected to train for self-directed adaptation and learn to sustain and improve their intellectual acumen. Moreover, learning functions as a consistent predictor of creativity and innovation [41]. Additionally, Rubenstein [42] emphasized the need to investigate creative performance in the context of HEIs. Likewise, Greene [43] urged scholars to examine whether creative performance is affected by learning outcomes in higher education.

Also, creative self-efficacy and leadership/supervisory support serve as antecedents of creative performance [44,45]. Thus, creative self-efficacy and leadership/supervisory support were investigated as predictors of creative performance based on the respective endorsements of Thundiyil [46] and Mathisen [47].

United Nations Development Programme (UNDP) titled “Human Development Indices and Indicators: 2018 Statistical Update” [48] provides a comprehensive statistical annex. This statistical update comprised data that give an overview of the state of development across the world, looking at long-term trends in human development indicators across multiple dimensions. UNDP’s Human Development Index (HDI) has delineated human progress, explaining information on people’s health, education, and income. Over the years, the HDI has served as a comparative tool of excellence and as a reliable platform for public debates on national priorities. Education indicators ranged across the systematic educational policies and practicalities which give a sense of overall national development. Henceforth, a comparison between China and Pakistan’s educational indicators for the year of 2017–2018 is compiled below in the shape of Table 1.

Likewise, in a report [49] from the United Nations Development Programme entitled “Uncertain time and unsettled lives: shaping our future in a transforming world”, Education [50], i.e., the Ministry of Education, Government of China statistics 2021–22 and Pakistan [51] economic survey have presented the following data in the shape of Table 2.

From this discussion, the following research questions were formulated to manage the inquiry:

Does EI affect learning outcomes (cognitive, social, self-growth, and satisfaction with the university experience) of the academics and administrative staff in HEIs of Pakistan and China?

Do self-directed learning (personal autonomy, personal responsibility, and personal growth) and KMPs (knowledge creation, acquisition, storage, sharing, and utilization) act as mediators between EI and learning outcomes?

Do learning outcomes (cognitive, social, self-growth, and satisfaction with the university experience) affect creative performance (creative self-efficacy and leadership/supervisor support)?

Does the comparative study exhibit identical/different results based on an assumed theoretical configuration?

## 2. Hypotheses Development and Conceptual Framework

### 2.1. Emotional Intelligence and Learning Outcomes

Emotional contagion theory affects learners heavily based on colleagues’ particular characteristics and their emotional expressions [52]. EI practice can be considered a critical asset to prompt and motivate individuals throughout Chinese and Pakistani educational institutes to maximize their learning outcomes [3,5]. Furthermore, the incremental theory of intelligence states that intelligence can be changed and increased, promulgated by positive emotions which help personnel foster and enhance their learning outcomes [53]. Emotions are regarded as a vital factor during the course of learning. However, it depends upon the learner whether or not they engage in their emotions, especially in Chinese and Pakistani HEIs, which can include motivating gestures or emotions that tend to discourage the employees. Therefore, EI can most definitely affect learning outcomes [3,5]. EI contributes to HEIs’ personnel in the realm of their learning outcomes that include social, cognitive, and self-growth outcomes.

Moreover, EI facilitates social interaction in an increasingly collaborative and shared learning atmosphere in HEIs [21]. Likewise, educational professionals with superior EI regularly utilize their emotional attributes and proficiencies to promote enhanced associations with others, thereby strengthening constructive shared connections [4]. Additionally, EI has a positive association with cognitive outcomes that enable individuals to interact constructively with peers and colleagues, which is vital for their cognitive expansion and development [54]. Similarly, critical reflection plays an essential role in self-growth outcomes that are predicated on the immediate encouragement of individualistic emotional experience. Therefore, individuals employing developed EI are more capable of assessing both personal and others’ emotions that facilitate better critical reflections, which means self-growth outcomes are fostered significantly [55]. Consequently, the hypothesis incorporating this type of intervention may be shaped as follows:

**H1.**  *There is a significant influence of EI on learning outcomes (cognitive, social, and self-growth outcomes and satisfaction with the university experience) of academics and administrative personnel.*

### 2.2. Emotional Intelligence, Self-directed Learning, and Learning Outcomes

Self-determination theory is a unique blend of intrinsic motivation and the interiorization of extrinsic motivation, which prompts personal choice/decision, optimum stimulus, constructive opinion, interactive engrossment, and self-salutation of the personal emotional state [56]. Self-directed learning, an antecedent of the self-determination theory, has revolutionized HEIs, as it highlights personal autonomy, personal responsibility, personal growth, and overall learning outcomes [57]. Moreover, research has examined and reported a positive association between EI and self-directed learning [5]. During this time, Greene [58] investigated phenomena, such as self-directed learning, to systematically assess the vital learning outcomes in higher education. Mega [59] proffered that individuals with optimistic emotions enrich their certainty in the incremental theory of intelligence, which considers intelligence as a variable that is extendable, thereby motivating individuals to expand their intellectual facilities via learning. Positive emotions further develop individuals’ trust in their emotional intelligence that motivates them to pursue learning. Hence, this type of motivation urges individuals to better comprehend their assigned tasks with a provision of autonomy in relation to scheduling, nursing, and regulating tasks on hand. Goleman [60] argued that EI is vital in relation to an individual’s success, even more so than IQ, since a person who favors learning through their own planned schedule learns with a higher level of proficiency than others. The author continued that self-assurance, flexibility, collaboration, and persistence for self-development, which are not measures of IQ, hold a place of prominence according to the appraisal of subjective learning outcomes. Self-directed learning in HEIs is a global phenomenon that manipulates or influences several practices such as goal formulation, metacognition, and self-assessment, all of which affect learning outcomes [5]. Consequently, the hypotheses are posited as follows:

**H2a.**  *There is a significant influence of EI on the self-directed learning (personal autonomy, personal responsibility, and personal growth) of academics and administrative personnel.*

**H2b.**  *There is a significant influence of self-directed learning (personal autonomy, personal responsibility, and personal growth) on learning outcomes (cognitive, social, and self-growth outcomes and satisfaction with the university experience) of academics and administrative personnel.*

**H2c.**  *Self-directed learning (personal autonomy, personal responsibility, and personal growth) mediates between EI and learning outcomes (cognitive, social, and self-growth outcomes and satisfaction with the university experience) of academics and administrative personnel.*

### 2.3. Emotional Intelligence, Knowledge Management Processes, and Learning Outcomes

In the information age, with the influence of knowledge-based theory, organizations such as HEIs have significantly focused on knowledge management as an important strategic resource that has been attributed to their survival and success [3]. Dogan and Vecchio [61] clarified the association between EI and KMP by explaining the quadrangular framework associated with a spherical procedure to acknowledge the present strategy formulation, response exploration, and feedback dissemination. Thus, EI is explained as a phenomenon that comprehends the influence and perceptiveness of emotions and utilizes aroused emotions as a source of connection [4]. Accordingly, personnel in the top tier of management need to evaluate and understand their own emotions and then make an effort to establish others’ emotions. Hence, the previously mentioned trickle-down effect will certainly pave the way for more effective KMP. Turnipseed and Vandewaa [62] argued that EI is positively associated with a helping behavior toward coworkers and, subsequently, allows EI personnel to influence KMPs in a positive manner. In general, there are three comprehensive purposes of knowledge management in organizations: expanding/polishing the organizations’ available knowledge; generating new knowledge or evolving innovations; and enhancing collaboration. Bose [63] posited that KMPs enabled organizations to augment the skill standards of personnel (these objectives apply to academic institutions as well). Thus, an organizational initiative toward knowledge management may consolidate the intended developments in KMPs and the employees’ subsequent performance and learning outcomes [38]. Consequently, the hypotheses may be shaped as follows:

**H3a.**  *There is a significant influence of EI on the knowledge management processes of academics and administrative personnel.*

**H3b.**  *There is a significant influence of knowledge management processes on learning outcomes (cognitive, social, and self-growth outcomes and satisfaction with the university experience) of academics and the administrative personnel.*

**H3c.**  *Knowledge management processes mediate between EI and learning outcomes (cognitive, social, and self-growth outcomes and satisfaction with the university experience) of academics and the administrative personnel.*

### 2.4. Learning Outcomes and Creative Performance

The componential theory of creativity defines emotional and social modules as obligatory for individuals to yield creative outputs, thereby underlining the significant characteristic of intrinsic motivation and the immediate influence of the organizational milieu on stirred motivation [64]. Therefore, creative performance is a shared/collaborative practice that involves interactive relations with immediate colleagues and partners and the immediate environment [65]. Wallas [66] investigated and claimed a four-phased creative problem-solving process in his pioneer work, which includes groundwork in pursuit of problem-solving, development of a precise line of action, comprehensive explanation of the course of action developed in the second phase, and authentication of the scheduled course of action through practical implementation. Parnes [67] explained a framework acknowledged as the Osborn–Parnes Creative Problem-Solving Model to supplement the aforementioned four-phased process. Additionally, the proposed framework included five phases: fact findings in the search for a problem encountered; a precise explanation of the problem in light of fact findings; idea realization to counter the existing problem; appropriate solution realization; and accepting the existing problem’s matured findings. Isaksen [68] later retitled the first two stages as problem finding and data finding.

On the other hand, the aforementioned frameworks have some shared characteristics (e.g., creative problem solving encompasses phases of producing ideas through a creative rationale and introducing cognitive practices to evaluate and implement the developed ideas linked with critical thinking) [69]. Certainly, the mentioned course of action instigates critical thought provocation, which is essential for creative problem solving [70]. Thus, a supplementary understanding of diverse measurements associated with creative and critical thinking may simplify the comprehension of the respective roles in problem-solving. Consequently, the following hypotheses are proposed:

**H4.**  *There is a significant influence of learning outcomes (cognitive, social, and self-growth outcomes and satisfaction with the university experience) on creative performance (creative self-efficacy and supervisory/leadership support) of academics and the administrative personnel.*

**H5.**  *There are significant differences in the effect of EI on learning outcomes, self-directed learning, and KMPs and learning outcomes on creative performance (creative self-efficacy and supervisory/leadership support) in Chinese and Pakistani HEIs.*

**Figure 1:**
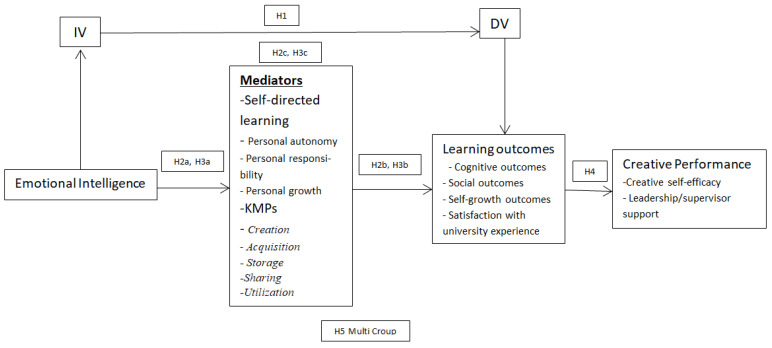
Conceptual Framework.

Conceptual framework is presented below in the shape of Figure 1.

## 3. Methods

Developed countries are guided toward economic and social development by HEIs that promote research and creativity [71]. This means research and innovative activities are essential for China and Pakistan’s economic and social development [12,13]. Therefore, the Higher Education Commission of Pakistan and China comprehensively emphasizes promoting research and developmental activities in HEIs [13,72]. Nonetheless, in the face of the mentioned urgency shown by respective Higher Education Commissions, measures in relation to research and developmental culture provided for HEIs’ customer satisfaction are quite insufficient [11,12]. Therefore, EI’s implementation in terms of training and developmental activities is inescapable to counter the contemporary educational challenges [5,15]. Hence, this study investigates the aforementioned research questions and tries to infer whether the research activities and measures taken for customer satisfaction of HEIs on behalf of academics and the administration are satisfactory.

### 3.1. Participants and Procedure

HEIs from different provinces were targeted for data collection (16 universities each from two provinces each of China and Pakistan). Provinces were Xi’an and Sichuan from China and Islamabad and Peshawar from Pakistan. In total, 1735 HEIs’ academics and administrative personnel from China and Pakistan were targeted for the data collection. The academics were composed of professors, associate professors, assistant professors, and lecturers, while the administrative staff consisted of departmental secretaries to front desk employees, according to their qualifications. HEIs’ personnel were contacted and consulted across the various departments (32 in total) in China and Pakistan. Furthermore, the data collection was acquired through face-to-face questionnaire administration, interviews, mail, and social networks across the provinces of Xian, Sichuan, Islamabad, and Peshawar. To date, numerous studies have used HEIs’ personnel samples involving EI [15,73]. The current study underwent a convenience sampling technique frequently utilized in EI investigations, often examining several countries [15,74]. The data collection was managed via a questionnaire disseminated to HEIs’ personnel utilizing electronic and physical distribution, administered in English. In both countries, the institutions’ deans, heads of departments, directors, and administrative personnel were informed about the study during individual appointments. Authorization was requested from the relevant stakeholders after that the dissemination of questionnaires between the intended sample of academics and administrative personnel were done.

Additionally, as previously mentioned, the present study combined self-administration, mail, social networks (e.g., WeChat, ResearchGate, and Facebook), and face-to-face interviews for the distribution of the questionnaires and data collection. According to Munir [74], mailed questionnaires are popular in developing countries like China and Pakistan; however, this has certain limitations in that the response rate may be low due to respondents’ busy schedules. Furthermore, face-to-face interviews were offered for individuals who found it problematic to complete the questionnaires manually. All participants were guaranteed confidentiality concerning the information provided. The demographics are listed in Table 3, while a table annexed in Appendix A explains the descriptive statistics for the considered instruments.

### 3.2. Selection of Measurement Instruments

Measurement items (89) were extracted from previously validated scales, as listed in Table 4. Some items underwent minor wording changes to fit a university setting. The questionnaire used a five-point Likert scale, with 1 indicating “strongly disagree” and 5 indicating “strongly agree.” Table 4 shows the sources of the measurement instruments.

## 4. Data Analysis

Smart PLS 3.2.7 was used to evaluate the hypothesized framework through structural equation modeling (SEM), which is regularly used for grounded investigations [83]. It is further vital to explain that SEM analysis has gained supreme preference while investigating management studies [31]. The covariance-based (CB-SEM) and PLS-SEM methods were used in the SEM method [84]. The PLS-SEM was selected over CB-SEM because it is better suited to exploring theoretical levels and complex relationships between latent constructs [74]. This method is appropriate for further investigating whether complex associations are present. It has extensively been utilized for theory testing and validation [85]. It assisted in scrutinizing the extent to which EI can anticipate learning outcomes from the respective professionals of HEIs. PLS further deals with any sample magnitude while establishing discriminant validity as a core advantage [74]. PLS, moreover, goes through two vital phases to examine the aforementioned framework (e.g., measurement model assessment, outer model evaluation, and structural model assessment) or inner model evaluation (e.g., the statistical configuration of latent variable associations).

### 4.1. Measurement Model Assessment

The measurement model consisted of EI, learning outcomes, and creative performance, including considered mediators that entail several items. Cronbach’s α value was ascertained to assess the reliability of the considered measures. Furthermore, composite reliability (CR) and convergent validity were calculated using average variance extracted (AVE). The assessed item loadings were more than the threshold value (0.7) presented in Table 5. Hence, α values and CR ascertained reliability.

Furthermore, AVE values evaluated convergent validity that should be more than the threshold value of 0.5 [84]. Table 6 confirms discriminant validity, as the square root of AVE is greater than the correlation between the selected constructs [85]. Furthermore, the single-factor test of Harman was used to weigh the common method variance that may influence cross-sectional studies. The result of the applied test was within the threshold value (lower than 50%); hence, the generated results were satisfactory. Moreover, multicollinearity concerns were not present because all variance inflation factors were within the threshold value (lower than five) [84].

### 4.2. Structural Model Assessment

PLS-SEM was utilized to assess the selected structural model after the statistical validation of the measurement model assessment. The hypothesized association was assessed through the bootstrapping technique with 1000 subsamples. Furthermore, the comprehensive structural model assessment was adhered to through path coefficients and coefficients of determination. Path coefficients are presented in Table 7 for hypotheses H1 to H4. Table 8, Table 9 and Table 10 investigated and explained mediation analysis (H2c and H3c) with specific indirect and total indirect effects.

Multi-group analysis (MGA) was applied to ascertain the potential differences in selected constructs among the considered countries. The possible differences may be the result of examined heterogeneity. Additionally, there may be unexamined heterogeneity that is not supposed to subsidize any or more than one selected variable [85]. Additionally, MGA was ascertained to examine the statistically significant path between Pakistan and China’s postulated frameworks before MGA endurance; the compositional invariance was investigated.

### 4.3. Hypothesis Testing

To determine the significance of hypothesized associations among emotional intelligence, self-directed learning, knowledge management processes, learning outcomes, and creative performance, a bootstrapping technique with 1000 subsamples was utilized independently in both countries’ samples. Table 7 hence presents the generated results for the significance of every hypothesized association. Furthermore, the eventual purpose of the applied statistical examination is to distinguish the differences among two nations’ sub-samples. Likewise, the variance determined, either distinctly or collected for the entire framework, was expressively different between the selected samples. Moreover, the R^2^ values for self-directed learning and KMPs in Chinese personnel were greater than those observed for Pakistani personnel. This validates the superior prognostic influence of self-directed learning and KMPs in the Chinese sample. Therefore, Chinese personnel exhibited a more promising inclination for self-directed learning and KMPs than the Pakistani sample.

### 4.4. Mediating Analysis

To test and interpret the mediation, the considered study incorporated hypotheses H2c and H3c to test the mediation of self-directed learning and KMPs between EI and learning outcomes. The method of indirect effects analysis was utilized to assess the assumed relationship of mediation [86]. Furthermore, bootstrapping with 1000 resamples for the targeted countries samples was tested; this resulted in total and specific indirect effects. Hence, due to the presence of significant direct and indirect effects, partial mediation is established in the Pakistani sample. However, full mediation is achieved in the case of the Chinese sample.

### 4.5. Multigroup Analysis

The established theoretical association relating to H6 determines the possible degree of variance across the samples from the Chinese and Pakistani HEIs. In order to infer the possible statistical differences for the samples across the countries, multi-group analysis was performed to ascertain the potential differences. Therefore, Henseler’s [87] approach was utilized through non-parametric partial least square multi-group analysis (PLS-MGA). PLS-MGA further differentiates the two countries [84]. Furthermore, measurement invariance is assessed through the composite models (MICOM) approach.

Moreover, configural and compositional invariance were observed in the PLS-MGA and it is presented through Table 11. Configural invariance describes that the data usage for both samples’ measurement assessment, structural assessment, and algorithm sets were the same across the selected countries. A further permutation method with a sample of 1000 (min) permutations was applied for compositional invariance at a 5% significance level. Compositional invariance equaled/compared the original value/score correlations c with correlations extracted through empirical distribution after utilizing thepermutation process (cu); furthermore, if c surpasses the 5% quantile of the permutation process (cu), then compositional invariance is established. Moreover, MGA is assessed to be significant if the compositional invariance is confirmed statistically. The further statistical steps are stopped if the compositional invariance is not found as per the law of the MICOM approach.

Table 12 represents the variances in paths across the selected samples. The values below 0.05 or above 0.95 illustrated significant differences across the selected samples. Only three paths exhibited significance in terms of generated results. The paths other than the aforementioned three paths were not significantly different in the selected samples, thereby partially adhering to H6.

## 5. Discussion

The current research aimed to investigate the influence of EI on learning outcomes (social, cognitive, and self-growth outcomes and satisfaction with the university experience) of academics and administrative staff from HEIs in China and Pakistan. This study further explored the indirect influence of self-directed learning and KMPs between EI and learning outcomes. Likewise, the study assessed the direct influence of learning outcomes on creative performance from Chinese and Pakistani HEIs. The outcomes of the study support the literature in multiple ways. This study supports the certainty of academics and administrative personnel’s EI to facilitate learning outcomes in HEIs of Pakistan. However, this ascribed relationship is contradictory in the framework of Chinese HEIs. The inferred findings, therefore, endorse a significant and positive association between EI and learning outcomes in the Pakistani context.

Furthermore, these results support previous findings by Asrar-ul-Haq [15] and Zhoc [5], with Zhoc [5] investigating the students’ EI in relation to their learning outcomes in Hong Kong HEIs. The sample results of the study for Chinese HEIs revealed an insignificant relation between EI and learning outcomes, hence contradicting the findings of Zhoc [5]. Moreover, the findings concerning the Chinese context of this study validate previous studies [88,89]. This contradictory note is further clarified through Goleman [90] arguing that if one does not have control over one’s emotional abilities, such as distressing emotions, and does not have empathy and effective relationships, being intelligent will not be of any significant importance. Similarly, Wolfe [91] explained that educators should be emotionally intelligent; otherwise, their action or inaction may impede learning outcomes.

Moreover, Kollontai [92] argued that learning outcomes in HEIs are negatively affected when mutual interactions are perceived to prompt varied emotional streams. Therefore, EI can only be developed in light of supporting relationships; otherwise, negative effects are largely evaluated on learning outcomes [93,94]. In other words, one must challenge one’s own practices to serve as a good role model and emotional coach to improve one’s own learning outcomes and, thus, others’ [95]. Additionally, it should be noted that EI is somewhat of a new concept in the Chinese context. Therefore, professionals in Chinese HEIs are less likely to be inclined to EI and its application in practical fields. Future studies may expound further in this regard, while Chinese HEIs should endeavor to train and regulate EI in their institutions. Overall, HEIs are considered initiators of trends that contribute to countries’ economic and competitive survival. Iqbal [12] proffered that HEIs, in the modern world, create value in an effort to remain competitive through their present knowledge and skills. Therefore, HEIs are striving to familiarize their workforce with EI to counter pressure situations and generate advances concerning possible mechanisms for learning outcomes. This process will enable professionals to counter or overcome every situation with the shield of EI and emerge as an efficient learner out of each dilemma [26].

Furthermore, the outcomes of the present study offer significant and realistic insight into the indirect impact of EI on learning outcomes through the mediating effects of self-directed learning. These findings also demonstrate that EI significantly and positively affects self-directed learning, thereby facilitating the enhancement of learning outcomes in HEIs of both China and Pakistan. The inferred findings are in line with the results of Zhoc [5]. Also, the findings of this study depict that EI leads to self-directed learning, which, in turn, increases learning outcomes [5,58]. Similarly, the findings of this research deduced EI’s significant positive and indirect effect on learning outcomes through KMPs, hence conferring a positive perception regarding the selected Chinese HEIs’ individual sampling. However, the results in the context of Pakistani HEIs reflected a negative relationship between KMPs and learning outcomes. The extracted negative output may be a result of certain challenges, and KMPs pose some challenges as part of their practical application in the HEIs of developing countries like Pakistan. In actuality, the major challenge is directed toward the researchers and administration as pertaining to the efficient management of available institutional knowledge potential [96]. Despite the enhanced importance of KMPs in HEIs in developing countries, the established and practiced strategies for KMPs’ implementation are insufficient and irregular due to the complex nature of the available knowledge management resources [97]. This is particularly true for countries like Pakistan [98]. Additionally, there are some other challenges in developing countries in the process of the implementation of KMPs: inflexible organizational hierarchy; structure; individualized organizational culture; absence of leadership support; lack of proper awareness regarding the KMPs reimbursements for individuals and organizations; and lack of a proper incentive structure for serving individuals [71,99]. Several researchers further investigated KMPs and established these problems as significant mediators between EI and learning outcomes with positive outputs [33,34,38,100].

Furthermore, the study found that learning outcomes (e.g., social, cognitive, and self-growth outcomes and satisfaction with the university experience) significantly contribute to creative performance, which includes creative self-efficacy and leadership support of internal customers (in this case, academics and the administration) [101,102]. Moreover, Tan and Md. Noor [103] emphasized the prominence of management sustenance to enhance individual and institutional outcomes. This requires managers to engender information that specifies what personnel should be seeking from their HEIs, colleagues, peers, and jobs [13]. Moreover, this continuous interaction and motivation tend to encourage employees to enhance their skills and experience for positive overall outcomes [41,104,105].

Lastly, the present study produced a multi-group analysis that demonstrated the differences between the two samples. However, the output did not establish partial measurement invariance for the three variables, thus indicating that compositional invariance could not be established. As per the MICOM method, if a compositional variance is not established, then the procedure for conducting further analysis is halted. This study, however, conducted MGA for readers’ understanding, keeping in mind that MGA is not expressive without compositional invariance confirmation.

## 6. Conclusions

This study has supported extant literature on EI through a comprehensive organization of learning outcomes by incorporating self-directed learning, KMPs, and creative performance in HEIs of selected countries. Furthermore, the findings reinforced the study’s framework, which has been rarely acknowledged by extant literature. Overall, HEIs’ professionals from China and Pakistan are eager to capitalize on the available opportunities to be creative performers capable of using a more precise application of EI. In the realm of EI, this paper has reinforced the theoretical application of EI and enhanced the overall understanding of learning outcomes along with self-directed learning, KMPs, and creative performance in the context of developing countries. The findings revealed the prominent contribution of EI and its influence on learning outcomes and on professionals from the Pakistani sample. Moreover, the previously mentioned variables explained the impact from the perspective of a particular country. Furthermore, since the research only compared Chinese and Pakistani professionals in HEIs, the findings can only be generalized to China and Pakistan.

HEIs are extensively meant to be service-oriented and competition-focused, implying that HEIs are increasingly staff (academics and administrative personnel)-focused. Additionally, the more HEIs admit to the needs of inducted personnel, the more profoundly they would be acknowledged and respected within the volatile educational spectrum [13]. The findings of this current study should prompt the HEI think tanks in China and Pakistan to reflect, plan, train, and develop personnel in accordance with the ever-changing educational hurdles.

### 6.1. Implications for Research and Practice

The present study expands the literature threefold in terms of research. Firstly, it extends the literature on EI and its valuable contribution to HEIs. Several previous studies have predicted EI’s role in HEIs by exploring its role concerning personnel burnout and learning outcomes, as well as cultural, demographic, and several other variables [5,15]. However, these extant studies focused only on the academics, administration, or the students. In contrast, the present study contributed to the prevailing research by investigating academics and administrative personnel in previously mentioned countries. The outcomes involving HEIs’ professionals offer an obvious comparison to clarify the differences in EI between China and Pakistan.

Similarly, EI has further demonstrated certain effects for academics and administrative personnel in both countries. Literature has shown the prominence of EI in forecasting learning outcomes, as emotionally intelligent personnel instruct and mentor students and generate overall professional surroundings conducive for enhanced learning (Chen, 2019). The findings likewise contribute to modern-day educational demands that are vital for evolving learning theories that ultimately enhance EI. Secondly, this study attempted to reinforce this particular aspect of learning and its potential necessity for HEIs. The extracted results further underscored the legitimacy of learning outcomes by elaborating on the intermediary role of self-directed learning and KMPs and provide a direct relationship between learning outcomes and creative performance resulting from EI, thereby building a comparative understanding of each narrated variable. Thirdly, the study extended the literature on the proposed framework of EI and learning outcomes by providing a comparative integration pertaining to HEIs’ personnel from emerging (China) and developing (Pakistan) countries. The findings, therefore, acknowledge a comprehensive understanding of the considered variables and expand the prevailing literature regarding EI, learning outcomes, and creative performance in these respective countries.

Consequently, the present study posits certain implications for HEIs and policymakers to improve guidelines that may enrich EI, learning, and the creativity of their professionals in Chinese and Pakistani HEIs. The findings illustrate that professionals from China and Pakistan exhibited optimistic intents to learn and expand their creativity with the efficient application of EI. Furthermore, HEIs must be consulted to ascertain appropriate academic, administrative, and pedagogical approaches to enhance their professionals’ EI, learning capabilities, successful execution of their personal ideas with authority, and proper care and utilization of available KMPs, as well as proper assistance from institutions regarding learning and creativity. Thus, HEIs in China and Pakistan should familiarize themselves with different and often novel academic, administrative, and pedagogical approaches that might involve training coaches and establishing an efficacious realm of professionals. Furthermore, in a quest to provoke and enhance a learning attitude and creative thinking specifically among Pakistani professionals, policymakers need to ascertain pragmatic and implementable policies at a governmental and institutional level to enhance EI and learning through providing proper training and occasional encouragement.

### 6.2. Future Research Guidelines

The limitations present in this study have led to possibilities for future research and developmental activities. First, the present study only reflected academics and administrative personnel from HEIs in China and Pakistan. It would be interesting to include a student sample from these respective countries by utilizing a conceptual framework. Second, the sample selection involving academics and administrative personnel from only China and Pakistan could be viewed as another limitation. The results may be interesting should the same framework be utilized for other developed and developing regions. Furthermore, the study measured EI as a predictor of learning outcomes; nonetheless, forthcoming studies may also study the involvement of additional personality traits. Additionally, to further discover and reinforce the theoretical foundation of EI in China and Pakistan, longitudinal studies may also be of great value. Lastly, forthcoming studies can examine the different mediators and even moderators to lengthen the conceptual framework. Regardless of the aforementioned limitations of the present study, it still offers valued proposals for educators, administrative professionals, policymakers, and government authorities that will possibly empower them to engage in productive choices to increase the EI of personnel and enrich the learning outcomes in China and Pakistan.

## Figures and Tables

**Table 1:** Education Indicators; Comparison of China and Pakistan Human Development Report (2017–18).

Indicator	China	Pakistan
Adult (15–24) Literacy % (2006–2016)	95.1	57.0
Female (2006–2016)	99.6	65.5
Male (2006–2016)	99.7	79.8
% with some secondary education age (25–above) (2006–2017)	77.4	37.3
Pre-Primary	84	72
Primary	101	98
Secondary	95	46
Tertiary	48	10
Primary School Dropout rate (2007–2016)	..	22.7
Public Expenditure on education (%age of GDP) (2012–2017)	..	2.8
Human Development Index (HDI) Rank	86	150
HDI Category	High	Medium

**Table 2:** Education Indicators; Comparison of China and Pakistan Human Development Report (2020–2021).

Indicator	China	Pakistan
Human Development Index (HDI)—2021	0.768	0.544
Expected years of schooling—2021	14.2	8.7
Mean years of schooling—2021	7.6	4.5
HDI rank—2020	82	161
HE enrollment—2021	2,863,712	1,964,000 **
HE institutions—2021	3012	233 **
HE teachers—2021	1,885,200	56,000 **
Literacy rate	96.8% (2018)	62.8% (2021)
Expenditure on Education—2021	20.25% of Govt. expenditure	1.77% of GDP

HE = higher education; ** Estimated.

**Table 3:** Demographics.

	China	Pakistan	Aggregation
Questionnaires delivered/sent	1000	735	1735
Questionnaire received	481 (65.98%)	248 (34.01%)	729 (42.01%)
Male Respondents	308 (64%)	145 (58.5%)	453 (62.13%)
Female Respondents	173 (36%)	103 (41.5%)	276 (37.8%)
Age- 20–29	169 (35.1%)	93 (37.5%)	262 (35.93%)
30–39	136 (28.3%)	65 (26.2%)	201 (27.57%)
40–49	113 (23.5%)	54 (21.8%)	167 (22.9%)
50–59	63 (13.1%)	36 (14.5%)	99 (13.6%)
Qualification—PhDs	312 (64.9%)	157 (63.3%)	469 (64.33%)
Masters	169 (35.1%)	91 (36.7%)	260 (35.66%)
Academics	312 (64.9%)	157 (63.3%)	469 (64.33%)
Administration	169 (35.1%)	91 (36.7%)	260 (35.66%)
Designation—Professor	63 (13.1%)	34 (13.7%)	97 (13.3%)
Associate Pro.	107 (22.2%)	56 (22.6%)	163 (22.3%)
Assistant Pro.	122 (25.4%)	65 (26.2%)	187 (25.6%)
Lecturer	22 (4.5%)	2 (0.8%)	24 (3.3%)
Administration	169 (35.1%)	91 (36.7%)	260 (35.6%)
Job Tenure—0–5	169 (35.1%)	89 (35.9%)	258 (35.4%)
6–10	134 (27.9%)	67 (27.0%)	201 (27.5%)
11–15	47 (9.8%)	35 (14.1%)	82 (11.2%)
16–20	70 (14.6%)	23 (9.3%)	93 (12.7%)
21–25	39 (8.1%)	24 (9.7%)	63 (8.6%)
26–30	22 (4.6%)	10 (4.0%)	32 (4.3%)

**Table 4:** Sources of Measurement Instruments.

Variable	Dimensions	No. of Items	Source
Emotional intelligence (EI)		33	Schutte, Malouff [75]
Self-directed learning (SDL)		10	Lounsbury and Gibson [76]
Knowledge management processes (KMPs)	Creation (C) Acquisition (A) Storage (ST) Sharing (SH) Utilization (UT)	6 3 4 4 3	Bontis, Crossan [77] Gold, Malhotra [78] Lin and Lee [79] Bontis, Crossan [77], De Vries, Van den Hooff [80] Lee, Lee [81]
Learning outcomes Creative performance (CP)	Cognitive outcomes (CO) Social outcomes (SO) Self-growth outcomes (SGO) Satisfaction with the university experience (SUE)	5 5 5 1 6	Zhoc, Chung [5] Zhoc, Chung [5] Zhoc, Chung [5] Zhoc, Chung [5] Wang and Netemeyer [82]

**Table 5:** Item Loadings, Reliability, and Convergent Validity for overall Chinese and Pakistani Samples.

			Overall Sample			China Sample			Pakistan Sample		
S. No	Dimension	Loadings	α	CR	AVE	α	CR	AVE	α	CR	AVE
	Emotional Intelligence		0.97	0.97	0.57	0.97	0.98	0.59	0.97	0.97	0.52
EI1		0.72									
EI2		0.73									
EI3		0.82									
EI4		0.72									
EI5		0.74									
EI6		0.71									
EI7		0.78									
EI8		0.70									
EI9		0.68									
EI10		0.81									
EI11		0.73									
EI12		0.75									
EI13		0.73									
EI14		0.72									
EI15		0.83									
EI16		0.82									
EI17		0.78									
EI18		0.72									
EI19		0.80									
EI20		0.83									
EI21		0.75									
EI22		0.80									
EI23		0.79									
EI24		0.68									
EI25		0.78									
EI26		0.80									
EI27		0.76									
EI28		0.81									
EI29		0.70									
EI30		0.71									
EI31		0.73									
EI32		0.77									
EI33		0.72									
	Learning Outcomes (Cognitive Outcomes)		0.91	0.93	0.74	0.93	0.95	0.79	0.83	0.88	0.61
CO1		0.82									
CO2		0.88									
CO3		0.85									
CO4		0.89									
CO5		0.83									
	Social Outcomes		0.90	0.93	0.73	0.92	0.94	0.77	0.85	0.89	0.63
SO1		0.85									
SO2		0.87									
SO3		0.84									
SO4		0.86									
SO5		0.83									
	Self-growth Outcomes		0.88	0.91	0.67	0.90	0.93	0.72	0.78	0.85	0.54
SGO1		0.75									
SGO2		0.82									
SGO3		0.87									
SGO4		0.81									
SGO5		0.83									
	Satisfaction with university experience		1.00	1.00	1.00	1.00	1.00	1.00	1.00	1.00	1.00
SUE1		1.00									
	Self-directed Learning		0.94	0.95	0.67	0.96	0.96	0.74	0.86	0.89	0.45
SDL1		0.81									
SDL2		0.84									
SDL3		0.82									
SDL4		0.81									
SDL5		0.82									
SDL6		0.83									
SDL7		0.80									
SDL8		0.87									
SDL9		0.77									
SDL10		0.78									
	KMPs										
	Knowledge Creation		0.89	0.91	0.65	0.91	0.93	0.70	0.82	0.87	0.53
CT1		0.85									
CT2		0.76									
CT3		0.79									
CT4		0.83									
CT5		0.81									
CT6		0.76									
	Knowledge Acquisition		0.83	0.89	0.74	0.84	0.90	0.76	0.77	0.86	0.68
AQ1		0.88									
AQ2		0.84									
AQ3		0.86									
	Knowledge Storage		0.86	0.90	0.71	0.88	0.92	0.74	0.78	0.86	0.61
ST1		0.81									
ST2		0.82									
ST3		0.88									
ST4		0.86									
	Knowledge Sharing		0.85	0.90	0.69	0.86	0.90	0.71	0.81	0.87	0.64
SH1		0.79									
SH2		0.88									
SH3		0.82									
SH4		0.83									
	Knowledge Utilization		0.80	0.88	0.71	0.81	0.89	0.73	0.72	0.84	0.64
UT1		0.84									
UT2		0.87									
UT3		0.81									
	Creative Performance		0.94	0.95	0.77	0.95	0.96	0.80	0.88	0.91	0.64
CP1		0.88									
CP2		0.89									
CP3		0.89									
CP4		0.85									
CP5		0.89									
CP6		0.86									

**Table 6:** Discriminant Validity (Fornell and Larcker Criterion).

	A	C	CO	CP	EI	SDL	SGO	SH	SO	ST	SUE	UT
**A**	**0.865**											
**C**	0.819	**0.806**										
**CO**	0.770	0.845	**0.860**									
**CP**	0.788	0.851	0.817	**0.882**								
**EI**	0.838	0.882	0.878	0.848	**0.760**							
**SDL**	0.826	0.884	0.885	0.886	0.903	**0.820**						
**SGO**	0.784	0.895	0.847	0.835	0.878	0.883	**0.823**					
**SH**	0.794	0.817	0.780	0.838	0.827	0.848	0.774	**0.836**				
**SO**	0.763	0.858	0.875	0.819	0.866	0.888	0.850	0.774	**0.855**			
**ST**	0.798	0.860	0.830	0.876	0.873	0.910	0.841	0.845	0.829	**0.845**		
**SUE**	0.802	0.727	0.715	0.722	0.780	0.745	0.753	0.704	0.714	0.734	**1.000**	
**UT**	0.736	0.774	0.750	0.811	0.807	0.793	0.771	0.800	0.777	0.795	0.653	**0.845**

Notes: The data in the diagonal (in bold) is the square root of AVE of the construct.

**Table 7:** Results of Structural Model Path Coefficient (Direct Relationships).

Overall Sample					Chinese Sample			Pakistani Sample		
Hypotheses	Relationship	β	T	*p*-Value	β	T	*p*-Value	β	T	*p*-Value
**Emotional Intelligence and Learning Outcomes**										
**H1a**	EI → LO	0.324	6.492	0.000	−0.018	0.582	0.561	0.541	10.102	0.000
**Emotional Intelligence, Self-directed Learning, and Learning Outcomes**										
**H2a**	EI → SDL	0.902	52.261	0.000	0.921	54.798	0.000	0.835	15.953	0.000
**H2b**	SDL → LO	0.347	6.451	0.000	0.123	2.760	0.006	0.467	7.125	0.000
**Emotional Intelligence, KMPs, and Learning Outcomes**										
**H3a**	EI → KMPs	0.922	65.626	0.000	0.951	90.608	0.000	0.808	11.814	0.000
**H3b**	KMPs → LO	0.184	2.721	0.007	0.576	9.489	0.000	−0.107	1.459	0.145
**Learning Outcomes and Creative Performance**										
**H5**	LO → CP	0.866	33.756	0.000	0.946	81.708	0.000	0.529	2.970	0.003

**Table 8:** Specific Indirect Effects (China sample).

	Indirect Path						
Hypothesis	Relationship	β	Relationship	Β	Mediation Effect β	t-Value	Decision
***H2c***	EI → SDL	0.921	SDL → LO	0.123	0.113	2.784	Supported
***H3c***	EI → KMPs	0.951	KMPs → LO	0.576	0.548	9.475	Supported

**Table 9:** Specific indirect effects (Pakistan sample).

	Indirect Path						
Hypothesis	Relationship	β	Relationship	Β	Mediation Effect β	t-Value	Decision
***H2c***	EI → SDL	0.835	SDL → LO	0.467	0.391	6.124	Supported
***H3c***	EI → KMPs	0.808	KMPs → LO	−0.107	−0.087	1.445	Rejected

**Table 10:** Summary of Mediation Results.

	China		Pakistan	
Relationship	β	*p*-Value	Β	*p*-Value
SDL → LO KMPs → LO	0.123 0.576	0.006 0.000	0.467 −0.107	0.000 0.145

**Note:** Bootstrapping (*n* = 500).

**Table 11:** Compositional Invariance.

		China vs. Pakistan		
Selected Constructs	Configurational Invariance	C	5% Quantile of Cu	Partial Measurement Invariance
EI	Yes	1.000	1.000	Yes
LOs	Yes	*0.991*	*0.997*	*No*
SDL	Yes	1.000	1.000	Yes
KMPs	Yes	*0.993*	*0.998*	*No*
CP	Yes	1.000	0.999	Yes

Note: Italic values disrupt the assumptions of measurement invariance between Chinese and Pakistani samples.

**Table 12:** Multi-group Analysis.

	Path Coefficients Diff. (China−Pakistan)	*p*-Value (China vs. Pakistan)
EI → LO	0.040	0.340
EI → SDL	*0.136*	*0.000*
SDL → LO	*0.383*	*0.000*
EI → KMPs	0.234	0.303
KMPs → LO	*0.267*	*0.078*
LO → CP	0.050	0.661

Note: Italic values show significant differences.

## Data Availability

The raw data supporting the results of this article will be made available by the authors, without undue reservation.

## Appendix A

**Table A1:** Descriptive Statistics for the Considered Instruments.

Descriptive Statistics’ Overall Sample					Chinese Descriptive Statistics				Pakistani Descriptive Statistics			
Dimensions	N	Mean	Std. Deviation	Variance	N	Mean	Std. Deviation	Variance	N	Mean	Std. Deviation	Variance
EI	729	4.2172	0.81180	0.659	481	4.0203	0.92979	0.865	248	4.2351	0.66114	0.437
CO	729	4.2941	0.88887	0.790	481	4.0345	0.98441	0.969	248	4.1516	0.74914	0.561
SO	729	4.2436	0.87694	0.769	481	4.0091	0.98839	0.977	248	4.1411	0.75774	0.574
SGO	729	4.3529	0.82372	0.679	481	4.1250	0.98482	0.970	248	4.3798	0.65713	0.432
SUE	729	4.2867	1.04242	1.087	481	4.1351	1.14402	1.309	248	4.4032	0.83854	0.703
SDL	729	4.2827	0.83830	0.703	481	4.1170	0.95658	0.915	248	4.3440	0.60367	0.364
C	729	4.2993	0.82098	0.674	481	4.1284	0.98048	0.961	248	4.2661	0.65558	0.430
A	729	4.2551	0.91728	0.841	481	4.1268	1.02485	1.050	248	4.3374	0.73440	0.539
ST	729	4.3107	0.86850	0.754	481	4.1393	1.00563	1.011	248	4.4042	0.62396	0.389
SH	729	4.2383	0.88147	0.777	481	4.0920	0.98820	0.977	248	4.3790	0.66771	0.446
UT	729	4.2872	0.85226	0.726	481	4.0506	0.98083	0.962	248	4.3804	0.67139	0.451
CP	729	4.3484	0.83443	0.696	481	4.2346	0.95731	0.916	248	4.4220	0.56092	0.315
Valid N	729				481				248			

EI = Emotional Intelligence, CO = Cognitive Outcomes, SO = Social Outcomes, SGO = Self-growth Outcomes, SUE = Satisfaction with university Experience, SDL = Self-directed Learning, C = Creation, A = Acquisition, ST = Storage, SH = Sharing, UT = Utilization, CP = Creative Performance.

**Table A2:** Items used to operationalize constructs.

**Emotional Intelligence**
I know when to speak about my personal problems to others
When I am faced with obstacles, I remember times I faced similar obstacles and overcame them
I expect that I will do well on most things I try
Other people find it easy to confide in me
I find it hard to understand the non-verbal messages of other people
Some of the major events of my life have led me to re-evaluate what is important and not important
When my mood changes, I see new possibilities
Emotions are one of the things that make my life worth living
I am aware of my emotions as I experience them
I expect good things to happen
I like to share my emotions with others
When I experience a positive emotion, I know how to make it last
I arrange events others enjoy
I seek out activities that make me happy
I am aware of the non-verbal messages I send to others
I present myself in a way that makes a good impression on others
When I am in a positive mood, solving problems is easy for me
By looking at their facial expressions, I recognize the emotions people are experiencing
I know why my emotions change
When I am in a positive mood, I am able to come up with new ideas
I have control over my emotions
I easily recognize my emotions as I experience them
I motivate myself by imagining a good outcome to tasks I take on
I compliment others when they have done something well
I am aware of the non-verbal messages other people send
When another person tells me about an important event in his or her life, I almost feel as though I have experienced this event myself
When I feel a change in emotions, I tend to come up with new ideas
When I am faced with a challenge, I give up because I believe I will fail
I know what other people are feeling just by looking at them
I help other people feel better when they are down
I use good moods to help myself keep trying in the face of obstacles
I can tell how people are feeling by listening to the tone of their voice
It is difficult for me to understand why people feel the way they do
**Learning Outcomes (Cognitive Outcomes)**
I feel good at dealing with unfamiliar problems
I always think creatively while dealing with unfamiliar problems
I always think analytically and critically while dealing with unfamiliar problems
I always view things from a broader perspective
I always try to develop in-depth knowledge and skills in my field
**Social Outcomes**
I always communicate effectively with others
I am good at understanding others with ease
I always prosper while getting along with people of different cultural and ethnic backgrounds
I love to work collaboratively with others
I always demonstrate leadership skills as and when needed
**Self-growth Outcomes**
I always try to manage my time more effectively
I always try to learn a new skill or knowledge by myself
I have an ability for critical self-reflection
I am an enthusiastic follower of lifelong learning
I always uphold my personal and professional ethics
Satisfaction with university experience
I am satisfied with my university experience
**Self-directed/Lifelong Learning**
I regularly learn things on my own
I am very good at finding out solutions on my own
If there is something I don’t understand, I always find a way to learn it on my own
I am good at finding the right resources to help me to do well
I view self-directed learning based on my own initiative as very important for success in life
I set my own goals for what I will learn
I like to be in charge of what I learn and when I learn it
If there is something I need to learn, I find a way to do so right away
I am better at learning things on my own than most others
I am very motivated to learn on my own without having to rely on others
**Knowledge Management Processes**
**Knowledge Creation**
My university workers constantly generate new ideas
My university workers adapt their work to meet customer requirements
Members of my team actively talk with each other and share knowledge
My department transforms individual knowledge to shared knowledge
Members of my department regularly share knowledge with other teams
My department regularly creates innovative processes
**Knowledge Acquisition**
Knowledge is obtained from students
Knowledge is obtained from employee
Knowledge is obtained from partners / other stakeholders (Media, Education, Communication, Agencies)
The department I work for uses the databases, repositories and information technology applications to store the knowledge for easy access by all lecturers
The department I work for uses various written devices such as newsletter, manuals to store the knowledge which capture from the lecturers
The department I work for has several publications to display the capture knowledge
The department I work for has several mechanisms to store the knowledge for patent and copyright
**Knowledge Sharing**
My university makes constantly updated information available to me
My university has systems in place that efficiently capture workers’ knowledge
My university is highly committed to research and development
My university does all it can to launch new products and services
There exist incentive and benefit policies for new idea suggestions in utilizing existing knowledge
There exists a culture encouraging knowledge sharing
Work flow diagrams are required and used in performing tasks
**Commitment to Learning (Learning Orientation)**
Our institution’s ability to learn is the key to our competitive advantage
The basic values of this institution include learning as key to improvement
The sense around here is that learning is an investment, not an expense
Learning in my institution is seen as a key commodity necessary to guarantee institutional survival
**Creative Performance**
This institution carries out routine tasks in resourceful ways
This institution comes up with novel ideas to satisfy customer needs
This institution is known for generating and evaluating multiple alternatives for customer problems
This institution have new perspectives on old difficulties
This institution provides methods for solving problems when existing answers are not a parent
This institution generates creative ideas for service delivery
